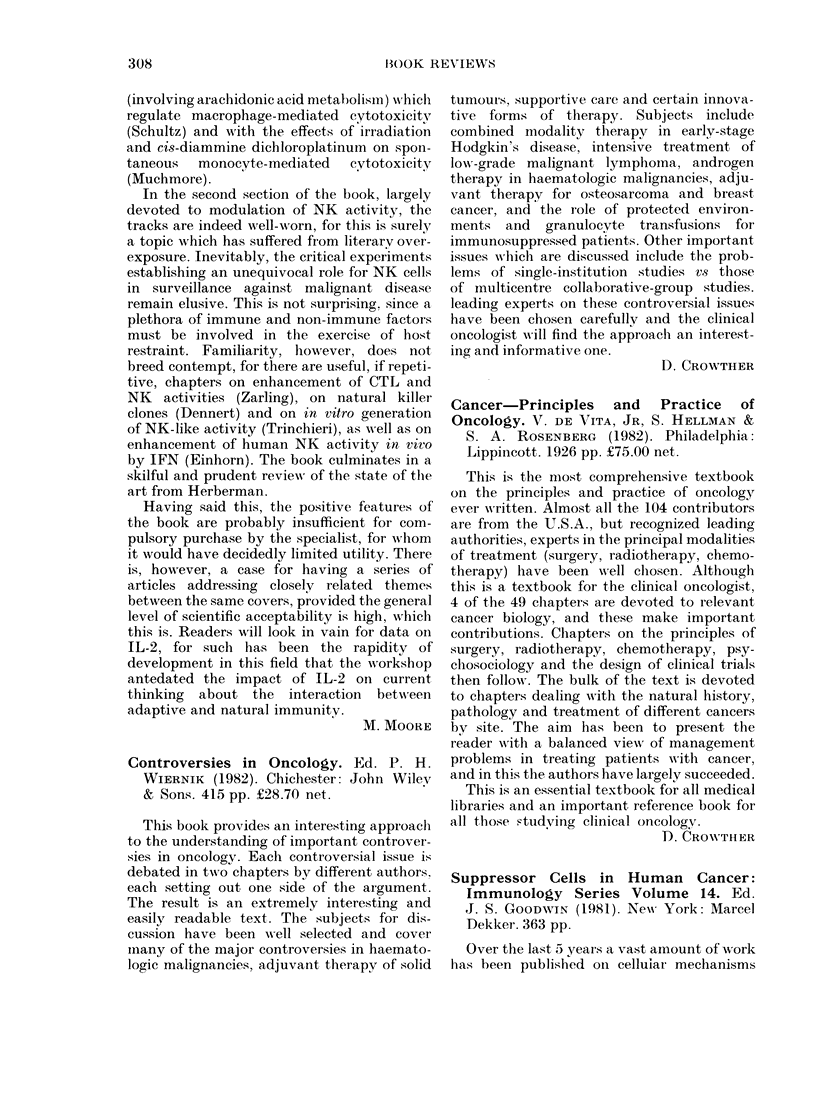# Cancer—Principles and Practice of Oncology. V

**Published:** 1982-08

**Authors:** D. Crowther


D. CROWTHER

**Cancer—Principles and Practice of Oncology.** V. DE VITA, JR, S. HELLMAN & S. A. ROSENBERG (1982). Philadelphia: Lippincott. 1926 pp. £75.00 net.

This is the most comprehensive textbook on the principles and practice of oncology ever written. Almost all the 104 contributors are from the U.S.A., but recognized leading authorities, experts in the principal modalities of treatment (surgery, radiotherapy, chemotherapy) have been well chosen. Although this is a textbook for the clinical oncologist, 4 of the 49 chapters are devoted to relevant cancer biology, and these make important contributions. Chapters on the principles of surgery, radiotherapy, chemotherapy, psychosociology and the design of clinical trials then follow. The bulk of the text is devoted to chapters dealing with the natural history, pathology and treatment of different cancers by site. The aim has been to present the reader with a balanced view of management problems in treating patients with cancer, and in this the authors have largely succeeded.

This is an essential textbook for all medical libraries and an important reference book for all those studying clinical oncology.

D. CROWTHER